# Building a Scaffolded Research Experience for Undergraduates

**DOI:** 10.3389/fpsyg.2019.00524

**Published:** 2019-03-19

**Authors:** Rachael D. Reavis, Margaret A. Thomas

**Affiliations:** Department of Psychology, Earlham College, Richmond, IN, United States

**Keywords:** undergraduates, pedagogy, scaffolding, publication, research laboratory

We teach at a small, teaching-focused liberal arts school on a semester schedule (teaching three courses per semester), with ~1,000 undergraduate students, no psychology graduate programs, and 18–25 psychology graduates per year. Faculty have some research expectations, which are partially confirmed through outside reviewers in the tenure process. However, our institution approaches faculty research as an opportunity to teach students, and we are rewarded for involving undergraduates in our research programs through tenure and internal grants.

Research is fundamental to our major and students regularly engage in research. The scaffolding, or framework, in our curriculum from lower-level courses to a cumulative senior research project, provides students the foundation needed to contribute meaningfully to research. Students have multiple opportunities to work closely with faculty. Increased student-faculty collaboration is associated with higher rates of graduate school attendance (Stoloff et al., 2015) and indeed Earlham is in the 95th percentile of institutions whose graduates complete psychology Ph.D. degrees (HEDS Baccalaureate Origins Report, 2017).

In lower-level courses, students do hands-on research, including replication projects and case studies, with 80% of our lower level courses having a research project. The most intensive research engagement comes through a three-course sequence: Statistics and Research Methods, an upper-level research-focused course, and Comprehensive Senior Research. Research suggests that integrated statistics and research methods education results in stronger performance and learning, both in the semester and at the end of the college career (Barron and Apple, 2014). Below, we talk about each of these classes in turn, before discussing the benefits of our curriculum for presenting and publishing with students.

## Scaffolding Research Opportunities

### Curriculum

Our research sequence begins with a Statistics and Research Methods combination course. Students are introduced to statistical concepts and sound research practices. They conduct small instructor-designed studies, using themselves as participants. The sophistication of the studies and written reports increases across the semester. Upon completion, students can articulate principles of good research design, select appropriate analytical tools, conduct analyses, and write a full-length APA-style report.

Next, students take a research-focused upper-level class, which they enter with the mental scaffolding, terminology, and skills associated with the process of research. We have multiple research courses, including Psychology of Prejudice and Behavioral Neuroscience. Each includes content information and requires students to conduct a research project. Projects vary across courses, but all involve students working in small groups (3–4) to design a content-related study, collect data through the subject pool (maintained by the psychology department), and present the results. These projects give students first-hand experience with a small research project of their choosing.

Group work and design constraints are important parameters for projects at this level. First, students support one another in groups; their skills and strengths are often complementary. Second, project constraints encourage positive experiences by keeping students within the scale of a doable project for novice researchers. Constraints include requiring only one independent variable, limited literature review breadth, and experience-appropriate expectations for independent statistical analysis.

When students reach the one-semester Senior Research course, they are prepared to articulate a research question, review the existing literature, design an appropriate study, analyze their data, and present their results in written and oral presentations. Although students work independently, faculty supervisors still provide scaffolding. Typically, two faculty supervisors lead the course, supervising 6–10 independent projects each. Faculty help students articulate sufficiently narrow research questions (usually quantitative) that can be reasonably addressed in one semester. Students receive detailed and ongoing feedback about their designs and writing and present their results to the department and visitors. Research suggests this type of course improves student learning and is viewed positively by faculty and students (Moore et al., 2018); anecdotally, we find the same. Students show considerable growth over the semester and are often proud of their accomplishments.

### Laboratory

Our structured curriculum supports research education for all students, but also provides strong preparation for high-quality Research Assistants (RAs) to pursue professional presentations and publication. Students receive elective credit as an RA, with the metric that 3 h of work per week during the semester is equivalent to one academic credit. Approximately half of each graduating class serves as an RA for at least one semester. Some RAs will pursue a lab-related topic for their senior project, but most will use the skills they learn in class and lab to pursue a topic in a different area of interest. Applications for RAs open in the mid-semester, which provides opportunities to a broader range of students and increases research participation (Wayment and Dickson, 2008).

The pace of research differs between the curriculum and our labs/research groups. In research courses, students complete an entire project in one semester, from idea to dissemination. In our labs, it is rare to move this quickly, particularly for research with children or non-human animals. The research in our labs is also more sophisticated and time-consuming than what students do in their classes. Specifically, we often have more conditions, more intensive recruitment strategies, longer protocols, and more complex statistical analyses. Given our small size and teaching focus, studies often take multiple semesters to complete.

Below we present two faculty research labs to demonstrate scaffolding, timelines, and support for meaningful undergraduate involvement in research. In the first lab, MT conducts research primarily with college students and adults using MTurk. In the second lab, RR conducts research with college students and children. In both labs each semester, students are given a syllabus-like document that clearly articulates the goals and tasks of each project, and the expectations for both faculty and students (Shanahan et al., 2015).

## Scaffolding Undergraduate Publication

### National Conference Presentations (MT)

The Social Fringe Lab averages four RAs per semester. My research projects involve straightforward protocols, allowing my lab to function well with returning RAs or frequent turnover. RAs build on their understanding of the research process, wherever they are in our curriculum. To start each semester, I have one or two projects ready for data collection and we finish one or two projects each semester. We spend the first 2 weeks of the semester reading background literature supporting the study, discussing the study design/method, and engaging in training on the protocol. Training for RAs involves learning software systems (e.g., Qualtrics, Sona-Systems), being a practice “participant,” leading the study with me as “participant,” and practicing with other RAs as “participants” while I observe. After training, RAs begin collecting data.

Once data collection is complete, we analyze data as a lab. I project my screen and we talk through the design and hypotheses of our study, then discuss which statistical tests are necessary before we analyze our data. At this point, I have RAs begin to work on a poster presentation. I share a template with them, assigning each RA a primary and secondary section. Each RA drafts their primary section, then provides feedback on their secondary section. In a lab meeting, I walk through the entire poster and we discuss edits. As a lab, we create an abstract for submission to a peer-reviewed national conference (which parallels the journal review process), as well as present research at an on-campus conference. Usually, my lab submits two abstracts per year.

After submission, RAs practice presenting the poster, regardless of the place of presentation (on- or off-campus). Practicing includes: a full walk-through of the poster contents, an “elevator speech” of the research, brainstorming questions that may be asked, and answering questions. In future semesters, if the abstract was accepted at a national conference, I coordinate with RAs who are still students to travel to the conference (as well as contact all RAs to update their CVs). At the conference, my RAs present the poster and I stand by to field questions they cannot answer from conference attendees. This poster serves as a draft for a paper, as it covers background literature, method, results, and discussion. Often, questions from conference attendees influence directions for future studies in that line of research, leading to student excitement at developing new studies for the project. Students who have worked on a project a long enough time (meaning over the course of multiple studies; recall my lab works well with high turnover) are invited to work on the manuscript as well.

### Publications (RR)

The Peer Lab is a comparatively larger lab in our department (although small by many standards), with approximately eight RAs each semester. There are typically three projects going on at any time: one with undergraduate participants and two with children (one conducted in the community and one conducted in the lab). Because the protocols with children are longer and working with children requires extensive training, it is important that most students work in the lab for multiple semesters. Many students join the lab early enough to stay 2–4 semesters (receiving academic credit each semester). Responsibilities in the lab are partially based on seniority in the lab and partially on where RAs are in the statistics/research methods curricular sequence (with most RAs entering the lab after the Statistics course, but usually before the independent senior project).

As in the broader curriculum, student work is scaffolded, with novice students being mentored by more senior students. Novice students generally begin running studies with straight-forward protocols. As students progress in the lab and in the curriculum, they take on more responsibility, working on project development, statistical analysis, and writing. This transition to independence is a transformative experience for students (Shanahan et al., 2015). In the Peer Lab, we spend several weeks each year discussing the replication crisis, p-hacking, and appropriate statistical and research practices.

During the fall and spring semesters, students work on conference posters and presentations, but much of the publication writing happens during the summer. One to three students work 40 h per week in the lab each summer for 4–6 weeks. This experience is paid and competitive, supported through endowed funds. Although some writing occurs during the academic year, the focus, and time during the summer allows for more intensive writing and feedback.

During the writing process, we work together on a rough outline. Students then generate search term lists that I approve and are assigned individual sections. They then generate a reading list. Students often need guidance on conducting thorough and appropriate literature reviews. Students will often find work that is decades old and need help discerning what is out of date. They then produce annotated bibliographies followed by integrated summaries. After several drafts and conversations, students begin writing the introduction and discussion, with the goal of a full draft by the end of the summer. Typically, students are heavily involved in the first submission only. Due to the long review times, students have often graduated by the time reviews come back. My practice has been to forward the reviews and get their approval for any revisions, but I make the revisions myself. Because of the intensive feedback and the students' lack of involvement in rewrites, I take first author position, with students following in order of contribution. My view here is that the scaffolding of the manuscript process continues into graduate school for those pursuing that path. Their experience with me is focused on the first submission. In graduate school and beyond, they will get ample experience with formal revisions. I view my role as a piece in a broader scaffolding process.

## Conclusion

Student successes in our labs are dependent on the broader curricular structure in our department. Students' experiences as RAs in our labs are both supported by and contribute to our broader focus on research competence across the major. Although we have had some success in generating publishable work with students during the semester, in our experience, institutional support for focused student research (such as paid opportunities during the summer or travel to conferences) is critical. We encourage departments to examine ways that the broader curriculum can support individual research labs and colleagues to create opportunities for students to build on their experiences in the classroom through meaningful contributions to our field.

## Author Contributions

RR and MT contributed equally to the writing and editing of the manuscript.

### Conflict of Interest Statement

The authors declare that the research was conducted in the absence of any commercial or financial relationships that could be construed as a potential conflict of interest.
